# Mesenteroaxial Gastric Volvulus: A Case Report

**DOI:** 10.31729/jnma.6519

**Published:** 2021-05-31

**Authors:** Sampanna Pandey, Madhav Paudel, Anuj Parajuli, Roshan Ghimire, Asmita Neupane

**Affiliations:** 1 Department of Surgery, Kathmandu Medical College Teaching Hospital, Kathmandu, Nepal; 2 Kathmandu Medical College Teaching Hospital, Kathmandu, Nepal

**Keywords:** *chest x-ray*, *gastric volvulus*, *polypropylene*

## Abstract

Gastric volvulus is defined as an abnormal rotation of the stomach. Classical textbook presentation may not always be present. Meticulous assessment and broadened differential diagnosis are thus crucial. Various types have been described in literature. Low threshold for detection with aggressive resuscitation and immediate surgical exploration on suspected incarceration or perforation are mandatory. We report a case of 16-years-female who had atypical presentation of mesenteroaxial gastric volvulus. Emergency exploratory laparotomy with wedge resection and primary repair of stomach with anterolateral gastropexy was performed. She had uneventful recovery with discharge on fifth postoperative day.

## INTRODUCTION

Gastric volvulus is a rare clinical entity. Its presentation varies and lead to significant morbidity and mortality when missed.^1^ Acute symptoms include pain of upper abdominal or lower chest with severe vomiting. The combination of pain, vomiting, and an inability to pass a nasogastric tube, known as Borchardt's triad, is present in 70% individuals.^1^ Patient with chronic or intermittent gastric volvulus has vague and often subclinical presentations, mild upper abdominal discomfort, bloating, non-bilious vomiting, early satiety, heartburn, and occasionally symptoms of pancreatitis.^2^

We present a case of 16-years-old female who presented with upper central abdominal pain associated with epigastric distension and non-bilious vomiting with significant-high amylase and lipase level who was diagnosed as gastric volvulus on computed tomography scan.

## CASE REPORT

We report a case of a 16-years-old female, presented with upper central abdominal pain, associated with upper abdominal distension and multiple episodes of non-bilious, non-blood stained vomiting containing food particles since 1 day without significant past medical or surgical history. On examination, her heart rate was 120 per minute, blood pressure 120/70mmHg and respiratory rate 20 per minute. Abdominal examination revealed epigastric distension and tenderness. Blood investigations revealed leukocytosis (21100) with significantly raised levels of amylase (1180) and lipase (1680).

**Figure 1.**
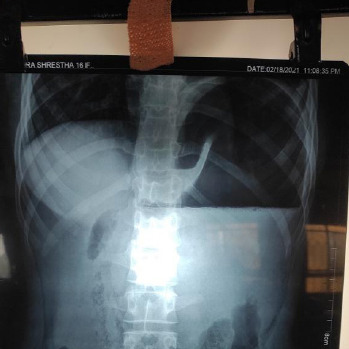
Abdominal X-ray of the patients revealed distended stomach with air fluid level.

The patient was admitted with a provisional diagnosis of acute pancreatitis and nasogastric decompression was done. On further evaluation chest X-ray (Figure 1) revealed a distended stomach with air-fluid level and as she did not have significant improvement with conservative management.

Then, contrast-enhanced computed tomography (CECT) of abdomen and pelvis was done which was suggestive of gastric volvulus. Immediate open surgical exploration was done under general anaesthesia.

**Figure 2.**
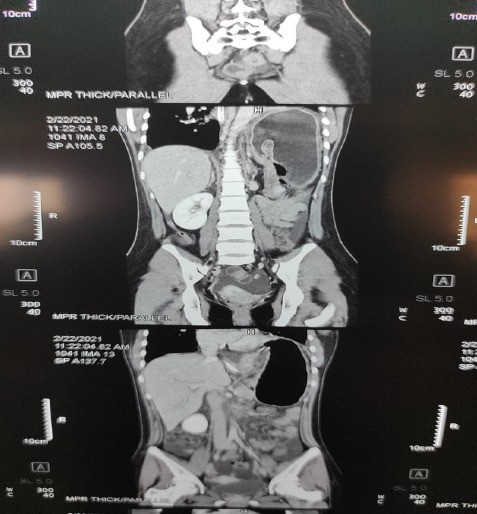
CECT abdomen and pelvis.

**Figure 3.**
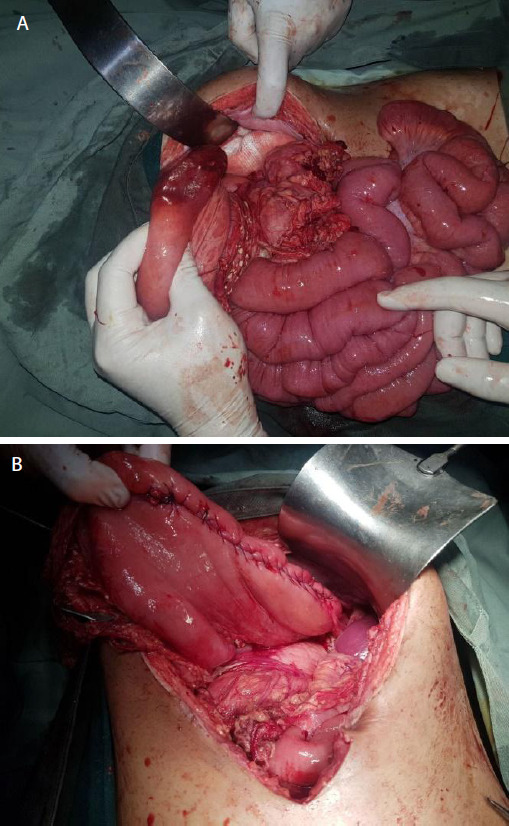
A) Mesenteroaxial volvulus with ischemia and gangrene over greater curvature B) Before closure.

Mesenteroaxial gastric volvulus with gangrene over greater curvature, wandering spleen and pulled up left hemidiaphragm was noted (Figure 3 A, B). Gastric volvulus was derotated, transverse colon mobilized, spleen placed in anatomical position and phrenicocolic ligament fixed to peritoneum with polypropylene 2-0 suture. Wedge resection of greater curvature with closure in 2 layers was done with polyglactin 2-0 suture. Anterolateral gastropexy with polypropylene 3-0 suture was done. The patient had an uneventful recovery. Sips were started on 1st postoperative day and liquids by 2nd postoperative day, nasogastric (NG) tube was removed on her 4th postoperative day and was discharged on 5th postoperative day.

## DISCUSSION

Gastric volvulus is characterized by rotation of the stomach along its long or short axis leading to variable degrees of gastric outlet obstruction, which may present acutely or chronically. Rotation of the stomach more than 180° causes complete gastric outlet obstruction; potentially, ischemia or strangulation of the stomach; and, potentially, necrosis, perforation, and abdominal sepsis. Mortality of acute gastric volvulus is high if not diagnosed and treated early.^3^

Gastric volvulus is rare with peaked incidence after the fifth decade with adults constituting 80 to 90 percent of cases.^4^ Gastric volvulus is characterized by abnormal rotation of the stomach along its horizontal or vertical axis. The stomach is normally maintained in position by several ligaments that fix the stomach to other abdominal structures and to the diaphragm, including the gastrocolic, gastrohepatic, gastrosplenic, and gastrophrenic ligaments preventing rotation.^3^

Gastric volvulus is classified as primary (idiopathic) or secondary depending upon its aetiology, organoaxial or mesenteroaxial according to the axis of rotation, and acute or chronic depending upon the clinical presentation. Primary (idiopathic) gastric volvulus is defined as volvulus due to abnormalities of the gastric ligaments like agenesis, elongation, or disruption of the gastric ligaments due to neoplasia, adhesions, or skeletal deformity.^5^ Secondary gastric volvulus occurs in two-thirds of patients with gastric volvulus and occurs due to other anatomic abnormalities, such as paraesophageal hernia, diaphragmatic hernia (Morgagni hernia, Bochdalek hernia, traumatic hernia), diaphragmatic eventration, or phrenic nerve paralysis, as well as anatomic abnormalities of other organs (eg, stomach, spleen).^3,4^ “Wandering spleen” (a mobile spleen only attached by an elongated vascular pedicle) and gastric volvulus have been described in association with a congenital diaphragmatic hernia, both conditions resulting from abnormal laxity or absence of peritoneal attachment due to congenital abnormalities.^6^

Organoaxial rotation occurs in 60% of cases, refers to rotation of the stomach along its long axis through a line that connects the gastroesophageal junction and the pylorus is associated with strangulation in upto 30% of cases.^3,5^ With mesenteroaxial volvulus, the stomach rotates around its short axis through a perpendicular line connecting the greater and lesser curvatures of the stomach. The rotation is usually partial (<180°) and is not generally associated with a secondary anatomic defect. A complex form of gastric volvulus combines elements of organoaxial and mesenteroaxial rotation.^3^

A diagnosis of gastric volvulus cannot be made with history and physical examination alone and plain radiographs or computed tomography (CT) are adjuncts. Acute symptoms include pain in the upper abdomen or lower chest associated with severe vomiting (which may become unproductive). The combination of pain, vomiting, and an inability to pass a nasogastric tube, known as Borchardt's triad, is present in 70 percent of patients with acute gastric volvulus. Hematemesis can occur due to mucosal ischemia or mucosal tears from vomiting.^3,5,7^ With complete gastric outlet obstruction, the stomach becomes dilated and fluid-filled, manifesting as upper abdominal distention with dullness to percussion. Auscultation may reveal gastric sounds in the chest. Signs of peritonitis (abdominal wall rigidity, rebound tenderness) may be present if significant gastric ischemia due to strangulation or perforation has occurred.^8^ Patients with recurrent vomiting may have electrolyte abnormalities, including hypokalemia or hypochloremic metabolic alkalosis, or evidence of volume depletion. Laboratory findings may also include an elevated white cell count, which may be due to inflammation, and acute stress and pain related to acute gastric distention. Other nonspecific laboratory findings may include elevated pancreatic enzymes and anaemia related to mucosal ulceration.^3^

The classic finding on plain abdominal radiograph is a single large, spherical gas bubble located in the upper abdomen or chest with an air-fluid level.^9^ A distinguishing feature of organoaxial volvulus is that the stomach lies in a horizontal plane when viewed on plain radiographs. Mesenteroaxial volvulus will have a spherical stomach on supine images but two air-fluid levels on upright films, with the antrum, positioned superior to the fundus. Prior placement of a nasogastric tube may dissipate the gastric bubble, but the course of the nasogastric tube will remain abnormal.^10^ CT of the abdomen or chest typically demonstrates a dilated stomach, often abnormally positioned in the chest. A swirl sign, in which the esophagus and stomach rotate around each other on transverse plane images, may also be evident. Findings suggestive of gastric necrosis include pneumatosis of the gastric wall, free air and fluid outside the gastric wall within the hernia sac, and lack of contrast enhancement of the gastric wall. CT also defines other anatomic abnormalities, such as diaphragmatic defects, and excludes other abdominal pathology as the source of symptoms.^10,11^

Initial treatment involves stabilizing the patient (fluid resuscitation and correction of electrolyte abnormalities) and immediate gastric decompression. Endoscopic decompression under the airway may be attempted if this fails. Immediate surgery is mandated in the following instances: inability to decompress the stomach with a nasogastric tube or endoscopic-assisted techniques (i.e. strangulation), gastric perforation or mediastinal contamination confirmed by imaging, shock or hypotension refractory to resuscitation, severe sepsis.^12,13^ The goals of treating patients with gastric volvulus are restoring the stomach to a more normal anatomic position, repairing any associated anatomic abnormalities, and preventing future stomach rotation. For unstable patients, an open approach is preferred. Once the gastric volvulus is reduced, the stomach should be examined for areas of ischemia. Decompression and derotation of the stomach results in rapid reperfusion. The majority of gastric necrosis and/or perforation occurs at the fundus, a location amenable to sleeve resection with a linear stapler. Partial gastrectomy or, rarely, subtotal gastrectomy may be needed; however, because of the robust circulation to the stomach, this is uncommon. If the entire stomach is necrosed and not viable, total gastrectomy without reconstruction with a jejunostomy tube and an esophagostomy is usually done. Patients are generally too ill to undergo reconstruction at this time. Once patients are stable and recovered, one can return electively for an esophagojejunal reconstruction or colon interposition.^12,13^ Stable patients may undergo either open or laparoscopic surgery. Following reduction and detorsion of the stomach, either surgical repair of the anatomical defect (for secondary volvulus) or gastropexy (for primary volvulus) can be performed.^14^
